# Primary Care or Primary Problem? Aligning Access Pathways with Patient Needs Across the Care Continuum

**DOI:** 10.3390/jmahp14020027

**Published:** 2026-05-01

**Authors:** Gregory J. Privitera, James J. Gillespie, Alexa Walton

**Affiliations:** 1 Department of Psychology, St. Bonaventure University, St. Bonaventure, NY 14778, USA; 2 Department of Business and Economics, Saint Mary’s College, South Bend, IN 46556, USA; jgillespie@saintmarys.edu; 3 School of Public and International Affairs, North Carolina State University, Raleigh, NC 27695, USA; ajwalto2@ncsu.edu

**Keywords:** healthcare access, multi-entry care, health policy, insurance, equity

## Abstract

In the United States, access to healthcare is shaped not only by patient need but also by payer policies that determine which providers are reimbursable, how care is sequenced, and what constitutes a legitimate entry point into the system. These gatekeeping functions, while valuable for supporting clinical prioritization, risk stratification, and continuity of care, can also unintentionally reinforce structural inequities and credential hierarchies that delay or limit timely and equitable care, particularly for historically marginalized populations. While reform efforts often focus on expanding benefits or provider networks, fewer address the underlying design of access itself or the rules that govern how patients enter care. It is argued in this paper that a more equitable and efficient healthcare system requires multi-entry care models, in which nurses, behavioral health clinicians, pharmacists, and community health workers may serve as condition-appropriate, reimbursable first points of contact within coordinated care teams. Drawing on evidence from Medicare, Medicaid, the Veterans Health Administration, and commercial payers, these models may support cost containment, improve care coordination, facilitate appropriate utilization, and promote earlier patient engagement. While findings from these models are not uniform across all settings, evidence suggests that outcomes are highly dependent on implementation context, system design, and supporting infrastructure. When implemented with appropriate safeguards (such as interoperable health records, team-based care requirements, and coordinated referral tracking), multi-entry systems can preserve continuity while expanding access. Payers are uniquely positioned to lead this transformation by aligning reimbursement policy with patient needs, supporting team-based care infrastructure, and embedding accountability into access pathways, thereby creating a system that can be more responsive, inclusive, and sustainable.

## 1. Introduction: Access Pathways Across the Care Continuum

In the United States, access to healthcare is shaped not only by patient need or clinical urgency but also by health insurance policies that determine when, where, and with whom care begins [1,2]. These policies do more than reimburse services; they shape and reinforce a cultural hierarchy of legitimacy within healthcare [3]. By designating which providers qualify for reimbursement and under what conditions, insurers function as gatekeepers, positioning primary care physicians (PCPs) as the default entry point, even when other service lines may be more appropriate or culturally aligned [4]. While intended to coordinate care, this gatekeeping can create bottlenecks that delay access, exclude behavioral and community-based expertise, and disproportionately burden underserved populations [5]. These outcomes highlight the need for reforms that challenge entrenched hierarchies and expand legitimate entry points. While coordinated entry through primary care has historically supported clinical prioritization, risk stratification, and continuity of care, these functions are not inherently tied to a single provider type. Rather, they reflect system design choices that can be achieved through multiple, coordinated entry pathways when supported by appropriate safeguards.

The cultural hierarchy of legitimacy reflects a social valuation of certain knowledge systems, professional roles, and care types, often favoring biomedical authority while marginalizing community-based and culturally informed practices [6]. Physicians hold the highest status, sustained by clinical training, institutional affiliations, and long-standing cultural prestige [7]. In contrast, nurses, community health workers, behavioral health specialists, and integrative practitioners face barriers to recognition and reimbursement, despite evidence of their effectiveness in patient-centered and preventive care [8]. These disparities persist through licensing laws, reimbursement policies, and institutional regulations that privilege certain expertise over others, limiting equitable access [9,10]. For clarity, this paper focuses on licensed healthcare professionals operating within established scopes of practice, regulatory oversight, and evidence-based clinical frameworks (e.g., nursing, behavioral health, pharmacy, and allied health professions). It does not extend to unregulated or non-evidence-based practices.

Notably, differences in clinical training are real and relevant, but they do not justify a uniform access rule that requires physician initiation for all care types. Leadership in care delivery is condition-dependent and increasingly distributed across teams with defined scopes and accountability mechanisms [11,12]. Clinical authority is taught, credentialed, and rewarded in ways that can reinforce biomedical expertise as the gold standard [13,14]. This prioritization can favor specialization over prevention and institutional legitimacy over lived experience, excluding perspectives essential to equitable, holistic care [15]. Patients whose backgrounds or beliefs differ from dominant paradigms often face systems that undervalue their perspectives, limit their choices, and compromise outcomes [16].

While this hierarchy remains entrenched [17], some private and for-profit insurers are recognizing the clinical and financial value of multi-entry care models [18]. Multi-entry care refers to a coordinated model in which multiple licensed providers can serve as reimbursable, condition-appropriate initial points of contact within an integrated care system. These models legitimize alternative entry points (e.g., community health workers, care coordinators, and social-needs screenings) through value-based payment reforms and alternative payment models [19,20]. Team-based approaches often improve outcomes, reduce unnecessary emergency visits, and better manage chronic conditions, yielding cost savings [21,22]. For example, Carelon Health reports that its integrated behavioral health program saves an average of $3060 per member annually, showing that expanding access through multi-entry models is not only sound policy but also good business [23].

It is argued in this paper that transforming healthcare’s cultural hierarchy is essential for equity in access and outcomes. This requires recognizing multiple knowledge systems, revising reimbursement policies that reinforce exclusion, and supporting non-physician providers as legitimate leaders in care delivery. The following sections examine credential hierarchies that constrain access, present the case for multi-entry, patient-centric models, analyze gatekeeping costs, and outline why payers are among the best positioned to lead policy reform through reimbursement redesign. Importantly, this paper does not argue that physicians lack expertise or should be displaced as clinical leaders. Rather, it examines how current access rules may overextend physician-first requirements beyond contexts where they are clinically necessary, and how coordinated, team-based models can preserve oversight while improving responsiveness. While prior work has examined team-based care and access expansion, this paper advances a policy-oriented framework that centers payer-driven access design as a mechanism for enabling condition-appropriate entry points across the care continuum.

## 2. Structural Hierarchies and the Cultural Cost of Credentialism

While often framed as clinically necessary, the dominance of primary care physicians (PCPs), particularly those with MD (Doctor of Medicine) credentials, as gatekeepers reflects a deeper hierarchy of credentialism rooted in institutional prestige and cultural norms [24,25]. Even Doctors of Osteopathy (DOs), despite receiving equivalent medical training with added emphasis on holistic care, are often viewed as less legitimate within the system, which is a perception shaped, in part, by hiring practices, insurance credentialing, and cultural narratives of what constitutes “real” medicine [24,26]. This same pattern affects other licensed professionals, such as nurse practitioners (NPs), physician assistants (PAs), clinical psychologists, social workers, addiction counselors, and community health workers (CHWs), who are often under-recognized despite evidence of their effectiveness in care delivery. Many of these providers face restricted reimbursement rates, lack the authorization to prescribe medicines, or are excluded from being designated as first-contact providers by insurers [25,27].

This credential-based stratification may, at times, reflect professional traditions and organizational design choices that do not always fully align with patient needs [28]. While some may argue that maintaining a high bar for clinical gatekeeping ensures quality, this argument overlooks the mismatch between provider training and patient need. For example, an MD is not necessarily better positioned than a licensed therapist to address anxiety, nor more trained than a registered dietitian in managing nutrition-related illnesses. Reimagining access therefore calls for a shift in cultural and institutional thinking about where care can begin, and with whom to lay the foundation for more flexible, patient-centric models that meet people where they are. Importantly, claims about cultural hierarchy should not be conflated with empirical evidence on quality or safety, which varies by condition, provider role, and care setting [29,30].

Despite ongoing efforts to expand coverage and provider networks, relatively less attention has been given to the structural design of access pathways themselves—specifically, how payer-defined entry points shape care utilization, timing, and equity. As healthcare systems continue to evolve toward value-based and team-oriented models, examining the role of aligning access pathways is both timely and necessary. This commentary addresses this gap by focusing on how access pathways can be restructured to better align with patient needs and system capabilities. It draws on a synthesis of evidence from peer-reviewed studies, program evaluations, and payer-reported outcomes across Medicare, Medicaid, the Veterans Health Administration, and commercial payer settings. These sources were selected to reflect a range of real-world implementations of team-based and multi-entry care models. While not intended as a systematic review, this approach allows for the integration of policy-relevant evidence to inform conceptual and structural considerations related to access design.

## 3. Patient-Centric Access: The Case for Multi-Entry Care Models

Patient-centered care requires that access pathways align with patient needs, clinical context, and available evidence rather than default professional hierarchies [12]. As patient-centric models gain traction, there is growing recognition that reorganizing access around patient needs rather than around provider credentials can offer a more responsive, inclusive, and efficient approach [12,31]. Multi-entry care models, which allow patients to initiate treatment with a diverse range of licensed professionals (e.g., NPs, psychologists, counselors, pharmacists, CHWs), align closely with patient-centered care. While physician-led coordination remains essential for many forms of care, multi-entry care models can provide a complementary care alternative by recognizing that patients are best served when they can engage directly with the provider most relevant to their concern [32].

The evidence supporting multi-entry care is growing. Nurse-led interventions support chronic disease management by enhancing self-care and quality of life, particularly for patients in underserved communities [33]. Pharmacists, when empowered to manage minor ailments and offer medication consultations, help streamline access, reduce unnecessary physician visits, and boost adherence [34], yet remain underutilized in primary care [22]. In the mental health space, video-based therapy offers comparable clinical outcomes to in-person care while improving patient engagement and lowering dropout rates [35]. These findings echo broader research suggesting that decentralized access points can support care delivery, facilitate engagement, and align more closely with patient needs when implemented within coordinated systems [36].

Expanding access through multiple entry points also introduces important considerations related to quality assurance and patient safety [37]. Without appropriate safeguards, risks may include misdiagnosis, delayed escalation of care, or fragmentation across providers. These concerns underscore that multi-entry models must be implemented within structured systems that include clearly defined scopes of practice, standardized triage protocols, interoperable health records, and coordinated referral pathways, supported by emerging models in integrated and team-based care. When these elements are in place, distributed access can enhance early engagement while preserving clinical oversight and continuity.

Some critics caution that primary care often serves as a central point of contact, and while its effectiveness in managing care pathways is not uniform across settings, allowing patients to bypass PCPs may increase the risk of fragmented care or duplication of services. Duplication refers to repeated or uncoordinated services (e.g., redundant diagnostics or parallel consultations) that can occur in the absence of shared information systems or coordinated referral pathways. Large-scale network analyses show that when care is distributed across uncoordinated providers, rather than streamlined through a central gatekeeper, patients can experience lower quality, higher costs, and reduced continuity of care [38]. However, such outcomes can overlook the potential of well-integrated systems (e.g., shared electronic health records, interdisciplinary care teams, and centralized communication tools) to ensure complementary care continuity regardless of where care begins. Critically, these safeguards must be robust, enforceable, and equitably resourced. EHR interoperability remains a known barrier, particularly when providers operate across fragmented health IT systems. To mitigate this barrier, payers and policymakers can invest in shared infrastructure, mandate interoperability standards, and incentivize participation in health information exchanges [39]. Still, while these safeguards are conceptually well-established, their implementation at scale remains uneven. Challenges related to interoperability, enforcement, provider participation, and regulatory alignment have historically limited the effectiveness of coordination mechanisms across fragmented systems [38]. As such, these safeguards should be understood not as guarantees, but as necessary conditions requiring sustained investment, oversight, and policy alignment to function effectively.

In practice, integrated electronic health records, shared care plans, and interdisciplinary team models can ensure continuity and communication across providers [40]. Patient-centric access also promotes autonomy and early engagement [41]. In contrast, forcing patients to interact with a gatekeeper they do not trust or cannot easily access can lead to disengagement, delayed diagnoses, and worsening outcomes [12]. Multi-entry care, then, is a realignment of the system with how people actually experience and navigate their health. Still, for these models to avoid unintended overutilization, appropriate guardrails should be built into reimbursement policy [42]. Unintended overutilization refers to increased use of duplicative, low-value, or non-indicated services that can arise when care pathways are poorly coordinated across providers or settings. Avoiding this includes service utilization caps, coordinated care protocols, and performance-linked incentives that reward integration over volume [43]. These are not limitations on access but protections for sustainability.

While evidence supporting multi-entry and team-based care models is growing, findings are not uniform across all settings or implementations [44,45]. Systematic reviews and program evaluations report mixed or context-dependent outcomes, particularly in systems where coordination infrastructure, reimbursement alignment, or workforce integration are underdeveloped [46,47]. These variations highlight that the effectiveness of expanded access models is contingent on implementation context, system design, and the presence of supporting infrastructure [48,49]. From this position, these models are not universally superior, but context-dependent approaches whose success varies based on system design, patient population, and implementation fidelity.

## 4. Equity and Efficiency: Addressing the Hidden Costs of Gatekeeping

Gatekeeping functions, when dictated by credential-based policies rather than clinical appropriateness, can introduce structural inefficiencies and reproduce disparities, and in some cases fail to support patient needs [50,51]. At the same time, it is important to acknowledge that access controls can serve protective functions when appropriately applied, particularly in complex or high-risk clinical scenarios. While earlier sections have emphasized how access is shaped by provider legitimacy and patient trust, this section shifts focus to the systemic mechanisms and consequences that make gatekeeping an increasingly costly and inequitable practice in health care.

First, from an efficiency standpoint, gatekeeping often inserts unnecessary administrative steps that delay care, particularly when PCPs are required to authorize access to services they do not directly deliver [52,53]. This misalignment between provider training and patient need (e.g., requiring physician approval for counseling, nutrition support, or substance use treatment) diverts time, increases costs, and impairs continuity. In Medicare Advantage, for example, more than 35 million prior authorization requests were submitted in a single year, many of which delayed access to clinically appropriate services that the approving physician would not personally deliver [53]. These inefficiencies are not merely administrative. In a national study of cancer patients, 94% reported experiencing delays due to prior authorization, and many described frustration, stress, and worsening symptoms while awaiting approval [52]. Federal audits further reveal that some Medicare Advantage plans issue denials for services that would have been approved under traditional Medicare rules, underscoring how these barriers can obstruct medically necessary care [54]. These ripple effects undermine coordination and divert resources from higher-acuity needs in already overburdened health care systems [30,55].

Second, rigid gatekeeping models artificially restrict the use of an already stretched workforce. Providers such as community health workers, pharmacists, and nurse-led clinics are not just adjuncts—they are vital care-delivery assets, especially in shortage areas [34,56]. However, reimbursement structures, referral requirements, and practical constraints (e.g., workflow pressures from high prescription volumes, insufficient staffing, and inadequate workspace, including a lack of private counseling areas) often prevent these professionals from operating at the top of their license [57]. At scale, these missed opportunities translate into workforce underutilization and lost savings, particularly in models where early intervention could prevent higher-cost care [58,59].

Third, while patient disengagement is often framed as an individual behavior, gatekeeping can act as a structural deterrent, particularly when it imposes access barriers (e.g., distrust, prohibitive costs, lengthy wait times, and institutional navigation challenges) that reduce patient satisfaction and engagement [60,61]. This deterrent is especially evident in behavioral health and chronic disease management, where missed referrals or long delays contribute to dropout and relapse [62]. In these cases, instead of protecting quality, gatekeeping defers care until it becomes more complex and expensive to treat [9,63]. For example, recent population-level analyses showed that U.S. counties with higher PCP workloads experienced significantly more days of poor mental and physical health [64], which further underscores the value of distributing care across mental health professionals and community-based providers. Still, there are legitimate concerns that expanding provider entry points could contribute to uncoordinated care or duplicative services, especially in under-resourced or fee-for-service environments lacking integration infrastructure [65,66]. These risks must be acknowledged and managed. A phased implementation strategy, coupled with shared clinical dashboards, team-based care mandates, and centralized referral tracking, can ensure coordination while preserving flexibility [67].

Finally, the current gatekeeping structure limits the scalability of proven interventions. Evidence-based programs involving pharmacists in chronic care, community health workers (CHWs) in social risk management, and nurses in care coordination consistently show strong outcomes [68]. However, when these services remain peripheral (requiring a PCP referral or excluded from reimbursement) they cannot be used at scale. For example, a Virginia-based chronic care management program incorporating pharmacists into team-based care demonstrated improvements in blood pressure and A1C control among enrolled patients [69], consistent with broader evidence that pharmacist-integrated care models improve diabetes outcomes and care coordination [70]. Still, the model’s long-term viability depended on tapping Medicare’s CCM reimbursement; a structural hurdle that blocked broader rollout [71]. In the face of inflexible funding rules and referral-based workflows, these interventions struggle to move from pilots into mainstream care.

The hidden costs of gatekeeping, then, are both human and structural: they can delay treatment, squander resources, and preserve hierarchies that no longer serve the system’s needs. By confronting these inefficiencies directly, stakeholders can begin to realign policy with performance by designing access structures that reward value, support population health, and reflect the diverse ways patients seek care, ultimately shifting from a system of exclusion and delay to one of inclusion and responsiveness. Critically, expanding access pathways must also be considered alongside broader concerns regarding the medicalization of everyday life. Lowering barriers to entry without clear guidance may increase demand for services that are not clinically necessary. To address this, access reforms should be paired with patient education, decision-support tools, and public health messaging that help individuals navigate care appropriately and engage the system in ways that align with clinical need [12].

## 5. Why Payers Are Among the Best Positioned to Redesign Access

The intent of this commentary is not to establish causal evaluation. The evidence cited includes peer-reviewed studies, program evaluations, and payer-reported outcomes, which vary in methodological rigor and generalizability. Accordingly, this analysis synthesizes policy-relevant evidence to inform a framework for strengthening access structures, rather than serving as a definitive empirical assessment.

While findings from payer programs and integrated care models are promising, they are not uniformly generalizable. Accordingly, the present analysis is intended as a policy-oriented synthesis rather than a definitive empirical evaluation. In a system where coverage defines access, payers (both public and private) are not merely financial intermediaries; they are architects of care pathways. Their decisions determine who qualifies as a reimbursable provider, how services are sequenced, and what counts as a legitimate entry point into care [72]. As such, insurers play a pivotal role in shaping both the clinical and cultural structure of U.S. healthcare. If healthcare is to move toward patient-centered, multi-entry care models, payers are among the best positioned to lead that transformation. As emphasized by the National Academies of Sciences, Engineering, and Medicine [73], payment policy is one of the most powerful levers to rebuild primary care as the foundation of a high-functioning system, especially when aligned with team-based, longitudinal, and accessible care. Because reimbursement policies determine which providers are recognized as eligible entry points, payer design decisions directly shape how and where patients enter the healthcare system. Table 1 presents a policy framework linking key access constraints to their underlying mechanisms, corresponding payer-level policy levers, and implementation safeguards. Taken together, these policy levers illustrate how access can be recalibrated without displacing physician leadership or compromising coordination and accountability.

Several real-world programs demonstrate that when payers loosen gatekeeping restrictions and authorize distributed care access, the result is associated with improved outcomes for patients. Humana’s Medicare Advantage plan, for example, implemented proactive, team-based care coordination without overreliance on traditional physician gatekeeping, leading to 23.2% lower medical costs and 30.1% fewer inpatient admissions among value-based care enrollees [74]. Likewise, within the Veterans Health Administration, each additional primary care visit resulted in an average annual cost reduction of $721 per patient, largely due to fewer emergency and acute care episodes, highlighting the fiscal benefit of enabling more touchpoints at the front end of care [75]. Similar outcomes were reported in Arkansas, where Blue Cross Blue Shield realized returns of $2.22 to $5.84 for every $1 invested in patient-centered medical homes and CPC+ (Comprehensive Primary Care Plus) programs, with financial gains driven by reduced inpatient and ED utilization [76].

To lead meaningfully, payers should go beyond pilot programs and commit to rewriting the reimbursement architecture that governs first-contact care. Today, many high-value providers (e.g., nurse practitioners, pharmacists, behavioral health clinicians, and CHWs) are excluded from initiating reimbursable care unless referred by a physician. This restriction often has limited supporting evidence and may reflect bureaucratic credentialing practices more than patient-centered care [39]. Payers have the policy authority and market leverage to overcome these barriers. Still, implementation will require careful planning to mitigate risks such as care fragmentation, data sharing gaps, and uneven capacity across provider types. These challenges should not deter reform but must be acknowledged and addressed through integrated models, shared infrastructure, and appropriate regulatory oversight. For example, insurers can require that all reimbursed providers participating in multi-entry models document care within unified health records, participate in regular care team huddles, and meet metrics for continuity. Additionally, value-based contracts should include safeguards to monitor against duplicative billing, excessive referrals, or low-value services, without reverting to blanket gatekeeping restrictions. While it is evident there is work to be done, following the status quo simply sustains known inefficiencies and undercuts the very foundation of equitable, quality care [73].

Some have already begun to do so. Aetna’s Accountable Care Organization model generated $29.25 in per-member-per-month savings, largely through integrated chronic disease management and flexible provider access, thereby demonstrating how embedding multi-entry care in value-based contracts can yield substantial payer returns [77]. Medicare’s patient-centered medical home demonstrations have also shown a positive return on investment, using per-member-per-month payments to offset startup costs and incentivize more inclusive care models [78]. These considerations reinforce that payer and policy design play a central role in structuring access in ways that balance flexibility with accountability.

Other reforms can support this shift. A national evaluation of Medicaid value-based payment reforms found that global budget models with quality-linked incentives led to better care and lower per capita costs—particularly when access points were diversified across provider types [79]. Meanwhile, a systematic review by Cattel and Eijkenaar [80] reinforced that combining global payments with entry-point flexibility produces strong returns by enabling timely, distributed care. These outcomes reinforce that access reform is not merely about inclusion; it is about precision by ensuring that the right provider meets the patient at the right time.

These examples show that payers are among the best positioned to lead and support access reform not just by reimbursing new provider types, but also by redesigning incentives to prioritize early engagement. Practical actions payers can take include:

- Reimbursing first-contact care from qualified non-physician providers;
- Removing prior authorization requirements for low-risk, high-value services;
- Eliminating or minimizing co-pays for distributed entry points in behavioral health and chronic care;
- Aligning value-based contracts with multi-entry workflows;
- Funding outcome-based pilot programs to scale what works;
- Requiring participation in shared health information exchanges and interoperable EHR systems to maintain continuity;
- Linking reimbursement to performance metrics (e.g., care coordination, patient experience, and avoidable utilization).

Skeptics may argue that expanding access through multiple providers invites fragmentation or overutilization. While these concerns are certainly valid and guardrails to ensure care coordination, data-sharing, and responsible utilization must be in place, when payers authorize access models that align with patient needs and delivery efficiency, they achieve meaningful cost savings and better care. At the same time, they build trust and satisfaction among members, especially those historically marginalized by rigid, physician-centered systems. Ultimately, taking action is not just good policy; it is good business. Value in healthcare should not be measured solely by what is restricted, but also by what is enabled [12]. By embracing this principle and advancing patient-centric, multi-entry care, payers can lead a national shift toward a U.S. healthcare system defined by access, responsiveness, and long-term sustainability.

## 6. Conclusions: Access, Expanded

Access represents a central structural component of U.S. healthcare, shaping equity, efficiency, and patient trust. Yet the prevailing model continues to restrict first-contact care through credential hierarchies and insurance rules that do not reflect clinical or cultural relevance. While many reforms have aimed to expand services, too few have redefined how and where care begins. Without addressing the gatekeeping mechanisms that dictate entry, efforts to expand access risk reinforcing the very disparities they seek to solve. It is argued in this paper that reimagining access requires confronting both policy architecture and the underlying values that shape it. As described in this paper, evidence from networks, including Medicare, Medicaid, the Veterans Health Administration, and commercial payers, suggests that enabling distributed care can yield cost savings, facilitate outcomes, and strengthen early engagement. Multi-entry models can therefore function as an equitable and efficient complement to physician-centered systems, expanding patient access where appropriate without displacing physicians’ central role in coordinated care.

What is needed going forward is a commitment to align reimbursement with outcomes and to remove or minimize the structural barriers that limit first-contact care from high-value providers to ensure the culture of care is oriented around what serves patients best instead of organizing care around tradition or professional hierarchies. This includes balancing access with accountability. Guardrails to ensure care coordination, data-sharing, and responsible utilization are not a threat to multi-entry models; they are a prerequisite for their success. By doing so, payers can not only expand access; they can help recalibrate the culture of U.S. healthcare more holistically around patient needs rather than institutional convention.

## Figures and Tables

**Table 1:** Policy framework linking access constraints, mechanisms, payer-level policy levers, and implementation safeguards.

Policy Element	Access Constraint	Underlying Mechanism	Policy Lever	Safeguards
Physician referral requirements	Physician initiation required regardless of clinical necessity	Can create access delays and unnecessary care sequencing	Allow direct access to licensed providers within scope	Standardized triage protocols; referral pathways
Prior authorization rules	Administrative approval required before care delivery	Can lead to bottlenecks and delays in treatment initiation	Implement condition-based prior authorization exemptions	Utilization monitoring; audit systems
Reimbursement restrictions	Limited reimbursement for non-physician first-contact providers	Can limit utilization of available workforce	Expand reimbursement eligibility across care teams	Defined scopes of practice; credentialing standards
Fragmented care coordination	Lack of integration across entry points	Can increase risk of duplication and discontinuity	Require participation in shared care models	Interoperable EHRs; team-based care requirements

## Data Availability

No new data were created or analyzed in this paper.

## References

[1] Last A. (2024). How did we end up with such an opaque and costly health care system?. The New Yorker.

[2] Medicaid and CHIP Payment and Access Commission (MACPAC) (2024). Prior Authorization in Medicaid.

[3] Todic J., Cook S.C., Spitzer-Shohat S., Williams J.S., Battle B.A., Jackson J., Chin M.H. (2022). Critical theory, culture change, and achieving health equity in health care settings. Acad. Med..

[4] Lazo-Porras M., Penniecook T. (2023). Health equity: Access to quality services and caring for underserved populations. Health Policy Plan..

[5] Primary Care Collaborative (2023). Health Is Primary: Charting a Path to Equity and Sustainability.

[6] Hardeman R.R., Karbeah J., Kozhimannil K.B. (2022). Applying critical race theory to policy analysis in the United States: The case of maternal mortality. Health Serv. Res..

[7] Samuriwo R. (2022). Interprofessional collaboration—Time for a new theory of action?. Front. Med..

[8] Hummel J., Feeley D., Lewis K., Rivera M. (2022). Better together: Coalitions committed to advancing health equity. Nurs. Outlook.

[9] Jindal M., Chaiyachati K.H., Fung V., Manson S.M., Mortensen K. (2023). Eliminating health care inequities through strengthening access to care. Health Serv. Res..

[10] Baars E.W., Hamre H.J. (2017). Whole medical systems versus the system of conventional biomedicine. Evid.-Based Complement. Altern. Med..

[11] Mitchell P., Wynia M., Golden R., McNellis B., Okun S., Webb C.E., Rohrbach V., Von Kohorn I. (2012). Core Principles and Values of Effective Team-Based Health Care.

[12] Privitera G.J., Gillespie J.J. (2025). Toward a Healthy Society: Comparative Perspectives on American Health Care Policy.

[13] Lawrence C., Mhlaba T., Stewart K.A., Moletsane R., Gaede B. (2018). The hidden curricula of medical education: A scoping review. Acad. Med..

[14] Rogers L., Hughes Spence S., Aivalli P., De Brún A., McAuliffe E. (2024). Quantitative survey measures assessing power dynamics in multidisciplinary teams. J. Interprof. Care.

[15] Lauwers E.D.L., Vandecasteele R., McMahon M., De Maesschalck S., Willems S. (2024). Patient perspective on diversity sensitive care. Int. J. Equity Health.

[16] Adeogun A., Faezipour M. (2025). Patient-centric paradigm: A systems thinking approach. Healthcare.

[17] American Medical Association (2024). Competition in Health Insurance: A Comprehensive Study of U.S. Markets.

[18] National Academies of Sciences, Engineering, and Medicine (2023). Federal Policy to Advance Racial, Ethnic, and Tribal Health Equity.

[19] Petrullo J. (2022). Survey illustrates high receptiveness from payers to alternative payment models. Am. J. Manag. Care.

[20] Center for Health Care Strategies (2024). Building a Health Equity Focus into Value-Based Payment Design. https://www.chcs.org/resource/building-a-health-equity-focus-into-value-based-payment-design-approaches-for-medicaid-payers/.

[21] Kangovi S., Mitra N., Norton L., Harte R., Zhao X., Carter T., Asch D.A. (2018). Community health worker support on outcomes. JAMA Intern. Med..

[22] Patel M.R., Phillips R.L., Peterson L.E. (2023). Pharmacists in primary care. J. Am. Pharm. Assoc..

[23] Carelon Health (2024). Integrated Behavioral Health Improves Outcomes. https://www.carelonbehavioralhealth.com/perspectives/integrated-behavioral-health-benefits.

[24] Bodenheimer T., Lo B., Casalino L. (1999). Primary care physicians as coordinators. JAMA.

[25] Bueter A., Jukola S. (2025). Multi-professional healthcare teams and epistemic injustice. Med. Health Care Philos..

[26] Kopelman H. (2023). Disparities in DO vs. MD Applicants to Subspecialties. KevinMD.com. https://kevinmd.com/2023/08/disparities-in-do-vs-md-applicants-to-subspecialties-identifying-challenges-and-bridging-the-gap.html.

[27] Harkless G., Vece L. (2018). Nurse practitioner reimbursement policy: Part one. J. Am. Assoc. Nurse Pract..

[28] Kaiser L., Conrad S., Neugebauer E.A.M., Pietsch B., Pieper D. (2022). Interprofessional collaboration and patient-reported outcomes. Syst. Rev..

[29] Barnett M.L., Balkissoon C., Sandhu P. (2022). Quality of care nurse practitioners provide. J. Am. Assoc. Nurse Pract..

[30] Ntais C., Kontodimopoulos N., Talias M.A. (2024). Gatekeeping or provider choice for sustainable systems?. J. Mark. Access Health Policy.

[31] Gillespie J.J., Privitera G.J. (2018). Patient-Centric Analytics in Health Care.

[32] Osmancevic S., Steiner L.M., Großschädl F., Lohrmann C., Schoberer D. (2025). Cultural competence interventions in nursing. Int. J. Nurs. Stud..

[33] Dou Y., Zhang L., Wang Y., Xu X., Wu B. (2025). Nursing care interventions in COPD management. Adv. Clin. Exp. Med..

[34] Dineen-Griffin S., Benrimoj S.I., Williams K.A., Garcia-Cardenas V. (2021). Pharmacist-led minor ailment service. BMC Health Serv. Res..

[35] Batastini A.B., Paprzycki P., Jones A.C.T., MacLean N. (2021). Videoconferenced mental health services. Clin. Psychol. Rev..

[36] Newman T.V., San-Juan-Rodriguez A., Parekh N., Swart E.C.S., Klein-Fedyshin M., Shrank W.H., Hernandez I. (2020). Pharmacist-led interventions in chronic disease management. Res. Soc. Adm. Pharm..

[37] Chen A.M. (2025). Re-evaluating access in healthcare: Focus on quality of care. Public Health Chall..

[38] Gebhart T., Fu X., Funk R. (2021). Go with the Flow? A Large-Scale Analysis of Health Care Delivery Networks in the United States Using Hodge Theory. arXiv.

[39] Torab-Miandoab A., Samad-Soltani T., Jodati A., Rezaei-Hachesu P. (2023). Barriers to health information exchange. BMC Med. Inform. Decis. Mak..

[40] Khatri R.B., Endalamaw A., Erku D., Wolka E., Nigatu F., Zewdie A., Assefa Y. (2023). Continuity and care coordination. BMC Health Serv. Res..

[41] Hickmann E., Richter P., Schlieter H. (2022). Patient engagement and empowerment. BMC Health Serv. Res..

[42] Bodaken B., Bankowitz R., Ferris T., Hansen J., Hirshleifer J., Kronlund S., Labby D., MacCornack R., McClellan M., Sandy L. (2016). Sustainable success in accountable care. NAM Perspect..

[43] McClellan M.B., Feinberg D.T., Bach P.B., Chew P., Conway P., Leschly N., Marchand G., Mussallem M.A., Teeter D. (2017). Payment reform for better value. Vital Directions for Health & Health Care.

[44] Bates S.M., Lin J., Allen L.N., Wright M., Kidd M. (2025). Can multidisciplinary teams improve the quality of primary care? A scoping review. EClinicalMedicine.

[45] Wong S.T., Krajnak J., Modayil M., Bull M., Hippe J., Beckmans M., Correia R. (2025). Team-Based Primary Care: Learning How to Evaluate the Outcomes and Implementation of Team-Based Care.

[46] Baxter S., Johnson M., Chambers D., Sutton A., Goyder E., Booth A. (2018). The effects of integrated care: A systematic review of UK and international evidence. BMC Health Serv. Res..

[47] Tsiachristas A., Stein K.V., Evers S., Rutten-van Mölken M. (2016). Financial incentives for integrated care: A systematic review. Health Policy.

[48] Shortell S.M., Wu F.M., Lewis V.A., Colla C.H., Fisher E.S. (2015). A taxonomy of accountable care organizations for policy and practice. Health Aff..

[49] Singer S.J., Burgers J., Friedberg M., Rosenthal M.B., Leape L., Schneider E. (2011). Defining and measuring integrated patient care: Promoting the next frontier in health care delivery. J. Gen. Intern. Med..

[50] Spalletta O., Green S. (2025). The gatekeeper’s dilemma. Sociol. Health Illn..

[51] Sripa P., Hayhoe B., Garg P., Majeed A., Greenfield G. (2019). GP gatekeeping impact. Br. J. Gen. Pract..

[52] Chino F., Baez A., Elkins I.B., Aviki E.M., Ghazal L.V., Thom B. (2023). Prior authorization in cancer care. JAMA Netw. Open.

[53] Freed M., Biniek J.F., Damico A., Neuman T. Medicare Advantage in 2024. KFF 2024. https://www.kff.org/medicare/issue-brief/medicare-advantage-in-2024-premiums-out-of-pocket-limits-supplemental-benefits-and-prior-authorization/.

[54] U.S. Department of Health and Human Services, Office of Inspector General (2022). State Requirements for Conducting Background Checks.

[55] Hall J. Medicare Advantage Prior Authorizations Can Delay Care. MarketWatch 2024. https://www.marketwatch.com/story/medicare-advantage-prior-authorizations-can-delay-healthcare-for-seniors-a-bipartisan-group-of-lawmakers-is-trying-to-streamline-care-900bdc80.

[56] Goode J.-V., Owen J., Page A., Gatewood S. (2019). Community-based pharmacy innovation. Pharmacy.

[57] Kambayashi D., Manabe T., Hirohara M. (2023). Pharmacists during COVID-19. BMC Health Serv. Res..

[58] Owens C.T., Baergen R. (2021). Pharmacy practice in community settings. Pharmacy.

[59] Valliant S.N., Burbage S.C., Pathak S., Urick B.Y. (2022). Pharmacists as accessible providers. J. Manag. Care Spec. Pharm..

[60] Kyruus Health (2023). Care Access Benchmark Report 2023.

[61] Velasco Garrido M., Zentner A., Busse R. (2011). The effects of gatekeeping: A systematic review of the literature. Scand. J. Prim. Health Care.

[62] Lindsay C., Baruffati D., Mackenzie M., Ellis D.A., Major M., O’Donnell C.A., Simpson S.A., Williamson A.E., Wong G. (2024). Missingness in primary care. BMC Med..

[63] Centers for Disease Control and Prevention (2022). Behavioral Health Services and Access Disparities.

[64] Privitera G.J., Gillespie J.J., Pamula A., Piper B.J. (2025). Physician workload and mental health outcomes. Popul. Health Manag..

[65] Dzau V.J., McClellan M., Burke S., Coye M.J., Daschle T.A., Diaz A., Frist W.H., Gaines M.E., Hamburg M.A., Henney J.E. (2017). Vital directions for health and health care. NAM Perspect..

[66] Hughes G., Shaw S.E., Greenhalgh T. (2020). Rethinking integrated care. Int. J. Integr. Care.

[67] Gatome-Munyua A., Sparkes S., Mtei G., Sabignoso M., Soewondo P., Yameogo P., Hanson K., Cashin C. (2025). Reducing fragmentation in primary care financing. BMJ Glob. Health.

[68] Kangovi S., Mitra N., Grande D., Long J.A., Asch D.A. (2020). Community health worker ROI. Health Aff..

[69] Virginia Department of Health (2026). Remote Patient Monitoring and Chronic Care Management. https://www.vdh.virginia.gov/heart-disease/programs/remote-patient-monitoring-rpm-chronic-care-management-ccm-and-transitions-of-care-toc/.

[70] Young M. Health System Adds Pharmacist to Improve Diabetes Care. Clinician 2024. https://www.clinician.com/articles/pharmacists-can-help-improve-diabetes-outcomes-in-the-community.

[71] Kadree M.A., Wiggins P., Thompson L., Warriner C., White M. (2025). Chronic care management model evaluation. Am. J. Public Health.

[72] Centers for Medicare and Medicaid Services (2025). Multi-Payer Alignment. https://www.cms.gov/priorities/innovation/key-concepts/multi-payer-alignment.

[73] National Academies of Sciences, Engineering, and Medicine (2021). Implementing High-Quality Primary Care: Rebuilding the Foundation of Health Care.

[74] Humana Inc (2023). Value-Based Care Benefits Patients and Physicians. https://policy.humana.com/news-and-resources/news-press/2023/value-based-care-benefits-patients-and-physicians--new-report.

[75] Gao J., Moran E., Grimm R., Toporek A., Ruser C. (2022). Primary care visits and cost. J. Prim. Care Community Health.

[76] Brown C.C., Tilford J.M., Berkemeyer A., Davis V., Whitlock A. (2020). Value-Based Primary Care.

[77] CVS Health (2018). Accountable Care Organizations. https://www.cvshealth.com/news/health-insurance/accountable-care-organizations-transforming-care-delivery-to.html.

[78] da Graca B., Ogola G.O., Fullerton C., McCorkle R., Fleming N.S. (2018). Patient-centered medical homes and costs. J. Ambul. Care Manag..

[79] Lewis A., Howland R.E., Horwitz L.I., Desai S.M. (2023). Medicaid value-based payments. JAMA Health Forum.

[80] Cattel D., Eijkenaar F. (2020). Value-based provider payment initiatives. Med. Care Res. Rev..

